# Mirror therapy for an adult with central post-stroke pain: a case report

**DOI:** 10.1186/s40945-018-0047-y

**Published:** 2018-02-23

**Authors:** Davide Corbetta, Elisabetta Sarasso, Federica Agosta, Massimo Filippi, Roberto Gatti

**Affiliations:** 1 Laboratory of Analysis and Rehabilitation of Motor Function, San Raffaele Scientific Institute, Vita-Salute San Raffaele University, Via Olgettina 60, 20132 Milan, Italy; 2 Neuroimaging Research Unit, Institute of Experimental Neurology, Division of Neuroscience, San Raffaele Scientific Institute, Milan, Italy; 3 Department of Neurology and Neuroimaging Research Unit, Institute of Experimental Neurology, Division of Neuroscience, San Raffaele Scientific Institute, Vita-Salute San Raffaele University, Milan, Italy; 4 Humanitas Clinical and Research Center, Humanitas University, Rozzano, Italy

**Keywords:** Stroke, Physical therapy modalities, Pain perception, Case reports

## Abstract

**Background:**

Treatment of central post-stroke pain (CPSP) after a thalamic-capsular stroke is generally based on pharmacological approach as it is low responsive to physiotherapy. In this case report, the use of mirror therapy (MT) for the reduction of CPSP in a subject after a stroke involving thalamus is presented.

**Case presentation:**

Five years after a right lenticular-capsular thalamic stroke, despite a good recovery of voluntary movement that guaranteed independence in daily life activities, a 50-year-old woman presented with mild weakness and spasticity, an important sensory loss and a burning pain in the left upper limb. MT for reducing arm pain was administered in 45-min sessions, five days a week, for two consecutive weeks. MT consisted in performing symmetrical movements of both forearms and hands while watching the image of the sound limb reflected by a parasagittal mirror superimposed to the affected limb. Pain severity was assessed using visual analogue scale (VAS) before and after the intervention and at one-year follow-up. After the two weeks of MT, the patient demonstrated 4.5 points reduction in VAS pain score of the hand at rest and 3.9 points during a maximal squeeze left hand contraction. At one-year follow-up, pain reduction was maintained and also extended to the shoulder.

**Conclusion:**

This case report shows the successful application of a motor training with a sensory confounding condition (MT) in reducing CPSP in a patient with a chronic thalamic stroke.

## Background

Stroke often causes impairment in movement control but can also affect perception [1, 2]. Alterations of stimulus integration are common after a stroke, with variable reported prevalence ranging from 11 to 85% [3], and sometimes these alterations of perception result in pain. Pain relates with the site of lesion and it is completely distinct from other painful conditions such as shoulder pain or spasticity [4]. It typically emerges from hemispheric lesions that involve the spinothalamic and thalamocortical pathways, leading patients to complain of sharping, stabbing, or burning through an experience of hyperpathia and allodynia [5, 6]. This association between sensory abnormalities and constant or intermittent central neuropathic pain, arising from damage of the sensory tracts, is known as the central post-stroke pain (CPSP) syndrome [7, 8]. The estimated incidence of CPSP comes up to 1 every 6 patients presenting a vascular lesion in the thalamus [8, 9], but its prevalence is difficult to estimate because of the co-occurrence of other painful conditions, such as spasticity or shoulder pain [4]. The pathophysiological mechanisms underlying the development of CPSP are thought to be related to the hyperexcitability or to the spontaneous discharge of damaged neurons located in the thalamus or in the cortex [10]. The CPSP syndrome is one of the less responsive conditions to physiotherapy treatment and it usually requires a pharmacological approach through the use of Amitriptyline, Gabapentin and Pregabalin [2].

Mirror therapy (MT), defined as the use of a mirror reflection of unaffected limb movements superimposed on the affected extremity, is often used to treat motor and perception problems [11, 12]. This technique was described for the first time in 1995 in studies reporting the reduction of phantom limb pain in arm amputees [13]; more recently, its use was described also for recovery of motor function after stroke [14, 15], for the treatment of complex regional pain syndrome type I [12] and other painful conditions (e.g., brachial plexus avulsion and after surgery) [16, 17].

This case report describes the beneficial effect of MT for the reduction of pain of the upper limb in a subject presenting CPSP in the left body side combined to sensory loss and mild movement disorders after a right haemorrhagic lenticular-capsular, thalamic stroke occurred five years before. To the best of our knowledge, the effect of MT for the treatment of CPSP has never been observed despite it has been defined deserving to be explored [11].

## Case presentation

### Case description

**Fig. 1:**
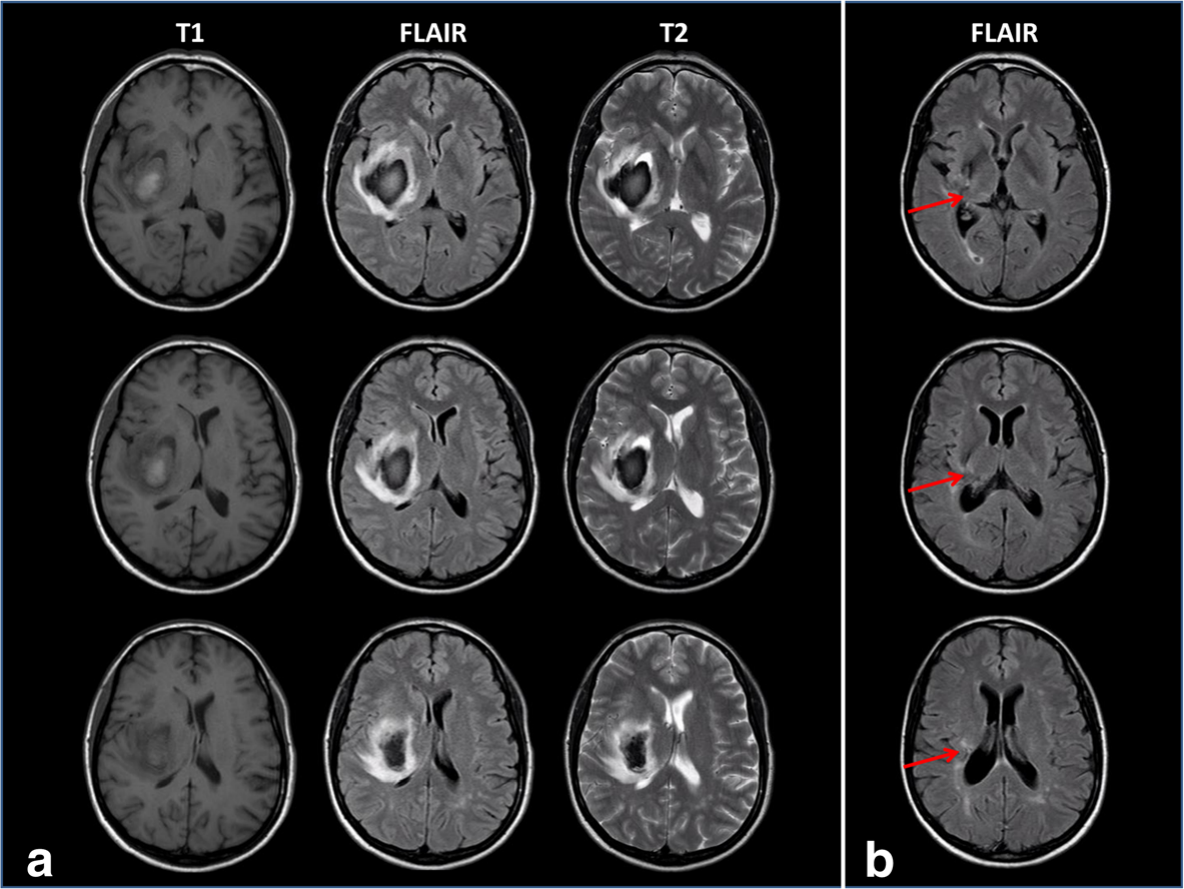
Magnetic resonance images showing the acute right lenticular-capsular-thalamic stroke lesion assessed with T1-weighted, Fluid-attenuated inversion recovery (FLAIR), and T2-weighted sequences (**a**); one-year follow-up MRI showing the lesion evolution assessed with FLAIR sequence. The red arrows show the posterior thalamic involvement (**b**)

The patient was a 50-year old, right-handed woman who experienced a haemorrhagic stroke in 2010. The computed tomography scan performed immediately after the acute event revealed a right lenticular-capsular, lateral thalamic and intraparenchymal hematoma, with a midline shift toward the left side. Two days after the acute event a magnetic resonance imaging scan of the brain confirmed the presence of this lesion (Fig. 1). After six weeks as inpatient for a rehabilitation program in our Institute, the patient entered a daily physiotherapy program for two months as outpatient, which led to a good recovery in motor control and strength.

Five years after the acute event, she returned to the Outpatient Service complaining of a CPSP syndrome, a persisting difficulty in the functional use of the left side of her body, especially of the lower limb during walking and stair climbing and a reduced postural balance. She was autonomous in everyday life activities (Functional Independence Measure = 120) [18], used a cane for walking outdoor, and needed augmented time for self-care.

The patient suffered from persistent allodynia and dysesthesia at her left upper extremity and at the left side of her face. The soft touch of an open hand was perceived as burning. Proprioception of the left upper and lower limbs was also impaired, as in the absence of vision she was unable to locate her left arm and leg. Three years before, she was prescribed Pregabalin to relieve pain (the patient does not remember the dosage), however she quit its use after few months because of its inefficacy. At the moment of the treatment she was not taking medications. At clinical examination, she presented low weakness at her left side (grade 4 out of 5 at Medical Research Council grading for strength of shoulder abductors, elbow flexors, wrist extensors, hip extensors, hip abductors, knee extensors, ankle dorsiflexors and plantarflexors) [19] and slight spasticity (modified Ashworth Scale grade 1) during the elbow and wrist extension; the knee flexion and the foot dorsal flexion [20]. She was able to perform isolated movements of the foot correctly and to move each finger of the left hand singularly.

### Intervention

**Fig. 2:**
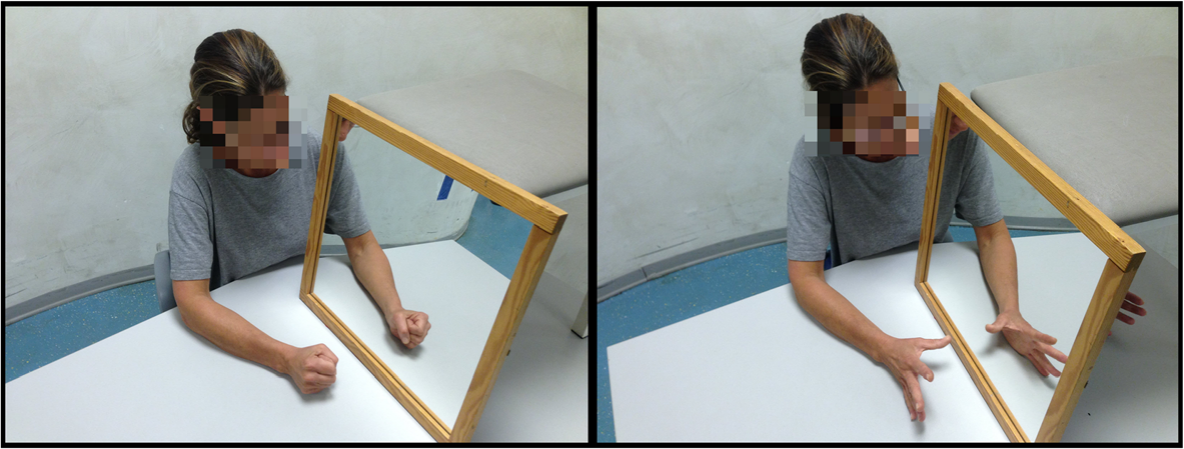
Example of upper limb movements performed by our patient during the mirror therapy training

The postural balance improved after few sessions of specific training on the treadmill and on instable surfaces, the patient achieved the ability to maintain the standing position on the left leg for some seconds. Afterwards, during one session targeted at improving coordination of the lower extremity, the patient performed exercises with a visual feedback provided by a mirror. After these exercises, the patient presented a positive good sensation at the leg, not related to movement, reporting that the leg was “*more sensitive*”. In the light of this unexpected finding, she was proposed to start MT in order to reduce pain at the upper extremity. The patient completed two consecutive weeks of MT training for five days a week. In each session, she was asked to perform symmetrical bilateral movements with the upper extremities while watching the image of the sound limb reflected by a parasagittal mirror superimposed to the image of the affected arm. Each session lasted 45 min. The requested movements were: forearm prono-supination, wrist extension and opening and closing the hand (Fig. 2). These movements were always proposed in a random order. Each movement was performed for 10 min at spontaneous speed (about one movement every second). Five minutes were spent for resting and for self-mobilization of the left arm and hand without the mirror. During the exercise, the patient was supervised by a physiotherapist. No further instructions, corrections or encouragements were given.

### Outcomes

Pain severity was assessed by the visual analogue scale (VAS 0-10 cm): the patient was asked to draw a vertical line on a horizontal 10 cm line, where she felt the pain intensity would be better represented, in a range from the left end of the line indicating “0 = no pain” to the right one indicating “10 = worst pain imaginable”. Hand and finger strength was assessed by the dynamometers Jamar and Pinch Gauge, and finger dexterity by the 9-Hole Peg Test [21]. The patient was evaluated at baseline, about one month before starting MT in two different occasions one week apart (to assess reproducibility), immediately before treatment and after treatment. VAS score was also obtained at one-year follow-up. VAS was used to assess pain severity at the hand and at the shoulder [22] in two separate conditions: at rest and during a maximal squeeze left hand contraction [23].

## Results

Before starting MT, the patient was stable as for functional use of the upper extremity and pain (Table 1). After MT training, the patient showed a reduction of 4.5 points at rest and of 3.9 points during the maximal voluntary contraction in the VAS score of the trained hand. The patient reported a reduction of pain intensity while burning sensation was still present, however the reduction of pain was described as a “significant relief”. A slight reduction of VAS score for shoulder pain occurred also, 1.2 points at rest and 2.3 points during the maximal voluntary contraction. Hand strength and dexterity did not show relevant variations after treatment, a difference of 3.7 N and 2.5 s from pre-treatment to post-treatment respectively reflect the difference observed during the two baseline assessments (Table 1).

**Table 1:** Clinical findings before and after mirror therapy

	Baseline 1	Baseline 2	Pre MT	Post MT	Δ pre-post	12 months follow-up	Δ pre-12 months follow-up
Shoulder pain (VAS, cm)	7.5	7.2	6.7	5.5	1.2	0.2	6.5
*Maximal Grip voluntary contraction*	8.8	9.1	9.6	7.3	2.3	0.2	9.4
Hand pain (VAS, cm)	5	4.6	5.3	0.8	4.5	1.5	3.8
*Maximal Grip voluntary contraction*	6.8	7.3	7.2	3.3	3.9	3.3	3.9
Hand Strength (N)	22	20	21.3	25	3.7	n.a.	
Finger Strength (N)	5	4	4.6	4.6	0	n.a.	
Dexterity (sec)	23"	20"	21.5"	24"	2.5"	n.a.	

MT: mirror therapy; ∆: Difference pre-post, difference pre-12 months follow-up; n.a.: not assessed. Baseline assessme nts were performed about one month before starting MT in two different occasions one week apart. Pain was assesse d with the Visual Analogue Scale (VAS 0-10 cm), strength was assessed with Jamar and Pinch dynamometers (values are expressed in Newton, N), and finger dexterity was assessed with the Nine Hole Peg Test (values are expressed in seconds)

The subject remained positively impressed by pain reduction after MT and autonomously decided to get a “mirror box” in order to continue the training at home.

One year later, relative to pre-treatment, the reduction of hand pain persisted both at rest and maximal voluntary contraction (Table 1). In addition, shoulder pain was further reduced (Table 1). It is worth noting that during the year, the patient performed spontaneously and steadily at home a similar MT training for the shoulder with flexion, abduction and external rotation with elbow flexed at 90°.

## Discussion and conclusions

This case report shows the application of MT on the upper extremity for the reduction of CPSP in a patient with a thalamic stroke occurred five years before. Findings from literature support the use of MT at least as add-on rehabilitation intervention for improving motor function in patients with stroke. The suppression of inappropriate proprioceptive input by visual information and/or the sensorimotor neural plasticity induced by MT may help motor recovery [12, 24]; furthermore, the application of MT in people with stroke presenting complex regional pain syndrome (CRPS) type I showed a significant effect on pain relief [25, 26]. In our patient, the MT was used with the aim to reduce CPSP occurring after a haemorrhagic thalamic-capsular lesion in a patient presenting pain and other sensory disturbances [7] rather than impairment of movement.

The pathophysiology of the CPSP is still unclear and different mechanisms involving the thalamus are suspected to underlie this phenomenon including deafferentation of ascending pathways (leading to sensory loss), disinhibition of its medial portion (leading to hypersensitivity), and abnormalities in spinothalamic function (leading to decreased or increased sensation of temperature, especially cold) [4]. These mechanisms are usually exacerbated by excitotoxic and inflammatory changes caused by the haemorrhagic lesion, resulting in a perception of pain even if it is not “activated” by noxious stimuli [27] (resulting in chronic pain). Furthermore, the altered balance between inhibition and facilitation of sensory-motor brain areas has been proposed as a possible underlying mechanism of central pain [4]. Particularly, a lesion of the lateral thalamus has been identified as one of the most common causes of CPSP [28]. One may speculate that in our subject the lesion of lateral thalamus and lenticular nucleus could induce an alteration in the functional connectivity between basal ganglia and primary/secondary somatosensory cortices which are involved in the sensory-discriminative dimension of pain, pain intensity perception and nociceptive information processing. According to previous findings, MT could optimize the altered balance between ispilesional and contralesional sensory-motor areas activation caused by the maladaptive reorganization of the somatosensory cortices, thus reducing pain perception [29, 30]. Another hypothesis explaining pain reduction provided by MT after limb amputation relies on a suppression of sensory discrepancies between vision and proprioception [11, 31]. In line with these findings, in our patient, after a short-term MT addressed to hand and forearm, the perception of pain changed. The brain continuously matches visual and kinaesthetic inputs during movements, linking what is seen with what is felt [32]. According to this hypothesis, the MT combination of visual illusion and movement would lead the CNS to reach a “sensory congruence”, which in turn would contribute to pain reduction [32, 33]. Interestingly, the patient’s awareness of the sensory illusion does not reduce the attempt of the CNS to achieve the sensory coherence between visual and proprioceptive information [34]. This would be in accordance with Ramachandran et al. [11] who suggested that pain mechanism is not influenced by the awareness of the trick.

The change of pain perception after MT extended also the shoulder, suggesting that the whole upper limb underwent a sensory re-organization. Nevertheless, a specificity of MT training and its long-term effect are suggested by the fact that the MT training addressed to the shoulder (spontaneously and steadily performed by the patient at home) specifically reduced shoulder pain at one-year follow-up.

Limitations are present; the observation of a single subject do not allow generalization: even if similar results are reported in studies enrolling people after stroke and presenting CPRP type I [25, 35]. During the treatment, the patient was asked not to change her lifestyle and not to introduce medications but the presence of co-interventions was only orally reported by the patient. The positive response to MT and the reporting of pain could have been influenced by the positive approach toward a new treatment. Lastly, a more comprehensive assessment of pain including other assessments than VAS would have improved the description of patient and of findings. In case of future trials assessing the effect of MT for reduction of CPSP, researchers should consider to include the assessment of neuropathic pain through the use of other instruments such as the painDETECT, the Douleur Neuropathique en 4 Questions (DN4), the Leeds Assessment of Neuropathic Symptoms and Signs (LANSS) or the Neuropathic Pain Scale [36, 37]. These questionnaires have high sensitivity and specificity for the detection of neuropathic pain, ranging from 83% to 85% for the sensitivity, for DN4 and LANSS respectively, and from 80% to 90% for specificity, for painDETECT and DN4 respectively [38].

In conclusion, this case report suggests that MT after stroke may affect the perception of pain resulting from CNS lesions and it could be considered as an additional approach to treat people presenting CPSP.

## Abbreviations

CNS: Central nervous system
CPSP: Central post stroke pain
CRPS: Complex Regional Pain Syndrome
MT: Mirror therapy
VAS: Visual analogue scale
